# Small Proline-Rich Protein 2A and 2D Are Regulated by the RBM38-p73 Axis and Associated with p73-Dependent Suppression of Chronic Inflammation

**DOI:** 10.3390/cancers13112829

**Published:** 2021-06-06

**Authors:** Xiangmudong Kong, Dan Wang, Wenqiang Sun, Mingyi Chen, Jinhui Chen, Jisen Shi, Jin Zhang, Xinbin Chen

**Affiliations:** 1 Comparative Oncology Laboratory, Schools of Veterinary Medicine and Medicine, University of California at Davis, Davis, CA 95616, USA; dkkong@ucdaivs.edu (X.K.); wangdan@njfu.edu.cn (D.W.); WQSUN@UCDAVIS.edu (W.S.); 2 Department of Pathology, University of Texas Southwestern Medical Center, Dallas, TX 75390, USA; mingyi.chen@utsouthwestern.edu; 3 Key Laboratory of Forest Genetics & Biotechnology of Ministry of Education of China, Nanjing Forestry University, Nanjing 210037, China; chenjh@njfu.edu.cn (J.C.); jshi@njfu.edu.cn (J.S.)

**Keywords:** P73, SPRR2A/SPRR2D, tumor suppression, inflammation

## Abstract

**Simple Summary:**

Small proline-rich protein 2A and 2D (SPRR2A and SPRR2D) are structure proteins of cornified cell envelopes and function as a protective barrier against diverse external insults. However, the role of SPRR2A/2D in chronic inflammation remains unclear. Here, we showed that SPRR2A/2D expression is controlled by a regulatory loop formed by RNA-binding protein RBM38 and tumor suppressor p73. We also found that RBM38-mediated expression of SPRR2A/2D was p73-dependent and that induction of SPRR2A/2D during keratinocyte differentiation was dependent on both p73 and Rbm38. Furthermore, We found that *Rbm38^−/−^;Trp73^+/−^* mice exhibited weak expression of SPRR2A/2D in multiple tissues and were susceptible to systemic chronic inflammation. Together, our data reveal that SPRR2A/2D are novel targets of the RBM38-p73 loop and contribute to p73-dependent suppression of chronic inflammation.

**Abstract:**

Small proline-rich protein 2A and 2D (SPRR2A and SPRR2D) provide barrier function in terminally differentiated stratified squamous epithelia through the epidermal differentiation complex. However, little is known how SPRR2A/2D expression is controlled and their role in chronic inflammation. Here, we showed that that SPRR2A/2D expression is controlled by a regulatory loop formed by RNA-binding protein RBM38 and tumor suppressor p73. Specifically, we found that SPRR2A/2D expression was induced by ectopic expression of RBM38 or p73 but suppressed by knockout of Rbm38 or p73. We also found that RBM38-mediated expression of SPRR2A/2D was p73-dependent and that induction of SPRR2A/2D during keratinocyte differentiation was dependent on both p73 and Rbm38. Additionally, we found that SPRR2A/2D expression was closely associated with p73 expression in normal and cancerous tissues. To determine the biological function of the RBM38-p73 loop potentially via SPRR2A/2D, we generated a cohort of wild-type, *Rbm38^−/−^*, *Trp73^+/−^*, and *Rbm38^−/−^;Trp73^+/−^* mice. We found that *Rbm38^−/−^;Trp73^+/−^* mice had a much shorter lifespan than that for *Rbm38^−/−^*—and to a lesser extent for *Trp73^+/−^* mice—but were less prone to spontaneous tumors than *Trp73^+/−^* or *Rbm38^−/−^* mice. We also found that *Rbm38^−/−^;Trp73^+/−^* mice exhibited weak expression of SPRR2A/2D in multiple tissues and were susceptible to systemic chronic inflammation, suggesting that decreased SPRR2A/2D expression is likely responsible for chronic inflammation in *Rbm38^−/−^;Trp73^+/−^* mice, leading to a shortened lifespan. Together, our data reveal that SPRR2A/2D are novel targets of the RBM38-p73 loop and contribute to p73-dependent suppression of chronic inflammation.

## 1. Introduction

p73 is a member of the p53 family of tumor suppressors, which include p53, p63, and p73. The p53 family proteins are transcription factors and can regulate a group of genes involved in cell cycle arrest and apoptosis [1]. The *TP73* gene maps a region on chromosome 1p36 that is frequently deleted in neuroblastoma and other tumors, and was initially thought to function as a tumor suppressor gene like *TP53* [2]. However, it was later found that *TP73*, unlike *TP53*, is not a classic Knudsen’s tumor suppressor gene [3]. *TP73* is rarely mutated in human cancers but found to be frequently over-expressed in several types of human cancer. These apparently contradictory data are likely due to the presence of multiple p73 isoforms with opposing functions [4]. Due to the usage of two different promoters, *TP73* is transcribed as two isoforms, the full-length TAp73 isoforms that exhibit tumor suppressor activity and the N-terminally truncated ΔNp73 isoforms that exhibit oncogenic activity [3,5,6]. Additionally, due to its C-terminal alternative splicing, the *TP73* gene expresses at least seven different isoforms (α, β, γ, δ, ϵ, ζ, and η) [7].

Studies from in vitro and in vivo models have shown that p73 has a broad range of functions in development, differentiation, reproduction, metabolic processes, genomic repair, senescence, angiogenesis, and tumor suppression [8,9,10]. For example, mice deficient in all p73 isoforms are runty and exhibit defects in neural development and multiciliogenesis [4,11,12]. Similarly, mice deficient in ΔNp73 isoforms are prone to delayed onset of moderate neurodegeneration [13,14] but do not develop tumors. By contrast, mice deficient in TAp73 isoforms show normal development but are prone to spontaneous and carcinogen-induced tumors, infertility, and accelerated aging [15,16]. These different phenotypes observed in total or isoform-specific *Trp73*-KO mice clearly suggest distinct functions of TAp73 and Np73 isoforms in development and tumor suppression. Thus, understanding the functions of various p73 isoforms and their network would help develop p73-based therapeutic strategies for cancer management.

RBM38 is an RNA-binding protein and has been implicated in multiple cellular processes, including cell cycle regulation and differentiation. We previously showed that RBM38 is a target of the p53 family proteins, including p73, and can in turn regulate p53 family proteins [17,18,19]. Indeed, RBM38 stabilizes p73 mRNA by binding to its 3′UTR [20]. Thus, RBM38 and p73 form a feedforward regulatory loop. To understand the biological function of RBM38, we generated a *Rbm38*-null mouse model and found that like *Trp73*-KO mouse model [21], *Rbm38^−/−^* mice are prone to spontaneous tumors and chronic inflammation [22]. As chronic inflammation is closely related to cancer development, we postulate that RBM38 cooperates with p73 to regulate chronic inflammation and tumor suppression. To this end, we identified small proline-rich protein 2A and 2D (SPRR2A and SPRR2D) as novel targets of p73, which may contribute to inflammation and tumor suppression regulated by the RBM38-p73 axis.

## 2. Materials and Methods

### 2.1. Mice

*Rbm38^−/−^* mice and *Trp73^−/−^* (on a pure C57BL/6 background) were previously generated by UC Davis mouse biology program [18,23]. To generate compound *Rbm38^−/−^;Trp73^+/−^* mice, two rounds mating strategies were used. First, *Rbm38^+/−^* mice were crossed with *Trp73^+/−^* mice to generate *Rbm38^+/−^*;*Trp73^+/−^* mice. Second, *Rbm38^+/−^*;*Trp73^+/−^* mice were mated each other to generate *Rbm38^−/−^;Trp73^+/−^* mice. All animals and use protocols were approved by the University of California at Davis Institutional Animal Care and Use Committee.

### 2.2. MEF Isolation

MEFs were isolated from 13.5-d-old embryos as described previously [24] and cultured in Dulbecco’s modified Eagle’s medium (DMEM) supplemented with 10% fetal bovine serum (Invitrogen, Carlsbad, Ca, USA), 55 μM *β*-mercaptoethanol, and 1× non-essential amino acids (NEAA) solution (Invitrogen, Carlsbad, CA, USA). To isolate WT, *Rbm38^−/−^*, p73^+/−^, and *Rbm38^−/−^*;*Trp73^+/−^* MEFs, *Rbm38^+/−^*;*Trp73^+/−^* mice were intercrossed.

### 2.3. Cell Culture, Cell Line Generation, and Differentiation

H1299, HCT116, SW480, and HaCaT cells were cultured in DMEM supplemented with 10% fetal bovine serum. RBM38-KO HCT116 cells were generated as previously described [25]. p73-KO H1299 cells were generated by using Crisp-Cas9 method. Briefly, H1299 cells were transfected with two gRNA-expressing vectors and selected with puromycin. P73 sgRNA expression vectors were generated as described previously [26]. The sequence for p73 sgRNA#1 is 5′ACC GCT TCC CCA CGC CGG CCT CCG 3′ and the sequence for p73 sgRNA#2 is 5′ CAC CGT CAA ACG TGG TGC CCC CAT C 3′. To induce HaCaT cells to undergo differentiation, HaCaT cells were cultured to confluent (~24 h) and then switched to DMEM containing 0.1% FBS plus 1.5 mM CaCl_2_ at indicated time.

### 2.4. Western Blot Analysis

Western blot procedures were as previously described [27]. Briefly, proteins were separated in 8–12% SDS PAGE gel and transferred to nitrocellulose membrane, which were then incubated with indicated antibodies. The signal was detected by enhanced chemiluminescence and visualized by VisionWorks®LS software (Analytik Jena, Upland, CA, USA). TAp73 antibody was purchased from Bethyl Laboratories and the actin antibody was from Sigma Aldrich. The RBM38 antibody was customized [17].

### 2.5. RNA Isolation, RT-PCR

Total RNA was isolated with Trizol (Invitrogen Life Technologies, Grand Island, NY, USA) reagent as according to the manufacturer’s protocol. cDNA was synthesized with RevertAid First Strand cDNA Synthesis Kit (Thermo Fisher ScientificTM, Carlsbad, CA, USA) and used for RT-PCR. The PCR program used for amplification was: (i) 94 °C for 5 min; (ii) 94 °C for 45 s; (iii) 60 °C for 45 s; (iv) 72 °C for 45 s and; (v) 72 °C for 10 min. From steps ii to iv, the cycle was repeated 22–30 times depending on the transcripts amplified. The forward primer used to amplify *ACTIN* was 5′- CTG AAG TAC CCC ATC GAG CAC GGC A -3′ and reverse primer 5′- GGA TAG CAC AGC CTG GAT AGC AAC G -3′. The forward primer for human *TAp73* was 5′- CAG ACA GCA CCT ACT TCG AC -3′ and reverse primer 5′- CTG CTC ATC TGG TCC ATG G -3′. The primers for human *RBM38* were 5′- CAA CGT GAA CCT GGC ATA TC -3′and 5′- TAA GTC CGC TGG ATC AAG GT -3′. The forward primer for human *SPRR2A* was 5′- CAG CTT CAG AAT TCA TCA GGA CCA A -3′ and reverse primer 5′- TGG GCA GAT TAC TGG CTA AGG A -3′. The forward primer for mouse *SPRR2A* was 5′- CAG TGC AAT CAG CCG TGC CG -3′ and reverse primer 5′- CCA GGC CAC ACT TGG GGA GG -3′. The forward primer for human *SPRR2D* was 5′- TTC AGG ATT CAT CAG GAG CAT GAG -3′ and reverse primer 5′- CAG GCA GGC CAC AGG TTA AGG AG -3′. The forward primer for mouse *SPRR2D* was 5′- TTG CCA GCC TCC ACC TGT GTG -3′ and reverse primer 5′- ATG AGG GAG GGG GAC ATG GCT -3′. The primers for mouse p73 were a forward primer 5′-AAG GGA CTA GCG AGG CAT CA 3′ and a reverse primer 5′-CCG GGG TAG TCG GTA TTG GA-3′. The primers for mouse actin were a forward primer, 5′-TCC ATC ATG AAG TGT GAC GT-3′ and a reverse primer, 5′-TGA TCC ACA TCT GCT GGA AG -3′. The primers for mouse *TNFa* were a forward primer, 5′-TGG CCT CCC TCT CAT CAG TT-3′ and a reverse primer, 5′-ACA AGG TAC AAC CCA TCG GC-3′. The primers for mouse *Rbm38* were a forward primer, 5′-GAC GCA TCG CTC AGA AAG T-3′ and a reverse primer 5′-GAG GAG TCA GCC CGT AGG T-3′. The primers for mouse *IL6* were a forward primer, 5′-GAG GAT ACC ACT CCC AAC AGA CC-3′ and a reverse primer, 5′-AAG TGC ATC ATC GTT GTT CAT ACA-3′.

### 2.6. Chromatin Immunoprecipitation Assay

ChIP assay was performed as previously described [28]. Briefly, 2 × 10^7^ cells were uninduced or induced to express *TAp73a* or *TAp73β*. Cell extracts were prepared with 2× modified buffer (1% SDS, 10 mm EDTA, 50 mmTris-HCl, pH 8.1, proteinase inhibitor mixture) and sonicated to generate 200–1000 bp DNA fragments. The protein-DNA complex was then incubated with 2 μg of anti-HA or mouse IgG at 4 °C overnight, followed by PCR analysis. The primers to detect the p53-RE in the SPRR2A promoter were a forward primer, 5′- GTA TTA TTC TCC CTG TTT ACA GTT C -3′, and a reverse primer, 5′- TCC TGT CTT ACA CCT TCC CTA -3′. The primers to detect the p53-RE in the SPRR2D promoter were a forward primer, 5′- AAG TAG AGG TAG AGT TGG GTT CA -3′, and a reverse primer 5′- TCT CAG TTT CAG TGA CTT TCG T -3′.

### 2.7. Histological Analysis

Mouse tissues or embryos were fixed in 10% (*w*/*v*) neutral buffered formalin, processed, and embedded in paraffin blocks. Embedded tissues were sectioned (8 µm) and stained with H&E.

### 2.8. Statistical Analysis

The Log-rank test was used for Kaplan–Meier survival analysis. Fisher’s exact test was performed for the statistical analysis. Values of *p* < 0.05 were considered significant.

## 3. Results

### 3.1. RBM38 Regulates SPRR2A/2D Expression via p73

**Figure 1:**
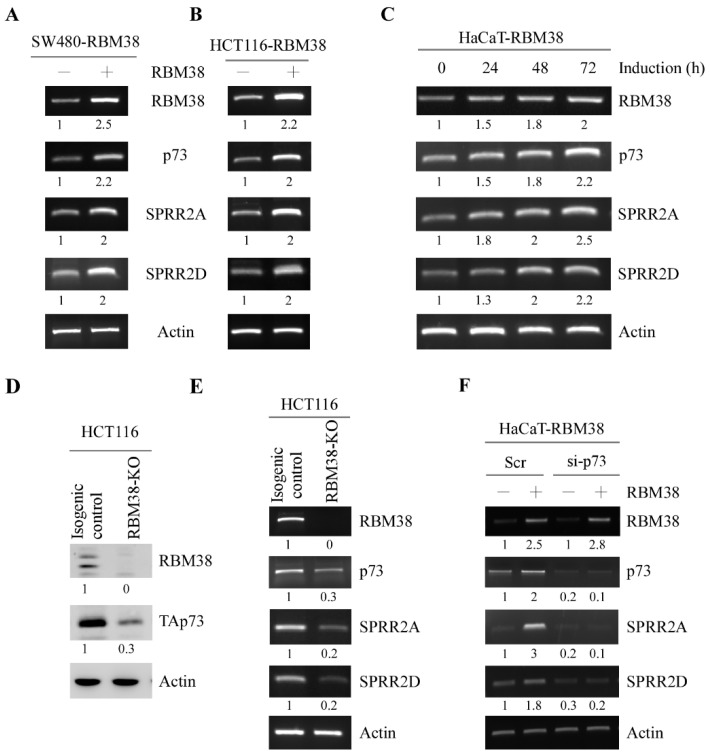
RBM38 regulates SPRR2A/2D expression via p73. (**A**,**B**) SW480 (**A**) and HCT116 (**B**) cells were uninduced or induced to express RBM38 for 24 h, followed by RT-PCR analysis to examine the levels of RBM38, p73, SPRR2A, SPRR2D, and actin transcripts. (**C**) HaCaT cells were induced to express RBM38 from 0 to 72 hours, followed by RT-PCR analysis to examine the levels of RBM38, p73, SPRR2A, SPRR2D, and actin transcripts. (**D**) The levels of RBM38 and p73 protein were examined in isogenic control and RBM38-KO HCT116 cells by Western blot analysis. (**E**) The levels of RBM38, p73, SPRR2A, SPRR2D, and actin transcripts were examined in isogenic control and RBM38-KO HCT116 cells by RT-PCR analysis. (**F**) HaCaT cells were transiently transfected with a control or p73 siRNA for 48 hours, followed with or without induction of RBM38 for 24 hours. RT-PCR analysis was performed to examine the levels of RBM38, p73, SPRR2A, SPRR2D, and actin transcripts.

Previously, we and others showed that p73-deficient mice are prone to spontaneous tumors along with chronic inflammation in multiple organs [4,21]. Interestingly, mice deficient in Rbm38 are also prone to spontaneous tumors with defects in immune system and have a short lifespan [22,25]. Since Rbm38 forms a feedforward loop with p73, it is likely that Rbm38 may cooperate with p73 to regulate a set of genes involving inflammation. To test this, RNA-seq was performed with MEFs or H1299 cells in that *Rbm38* or *Trp73* was knocked out. We found that small proline-rich protein 2A and 2D (SPRR2A and SPRR2D) were regulated by both RBM38 and TAp73. SPRR2A/2D are cornified envelope proteins and known to be involved in inflammatory diseases of the skin [29,30]. To further test this, the levels of SPRR2A/2D transcripts were measured in human SW480 and HCT116 cells that can inducibly express RBM38. We showed that upon induction, the level of RBM38 transcript was increased as expected along with an increased level of p73 transcript (Figure 1A,B, RBM38 and p73 panels), consistent with a previous report [20]. We also showed that the levels of SPRR2A/2D transcripts were increased by RBM38 in both SW480 and HCT116 cells (Figure 1A,B, SPRR2A and SPRR2D panels). Similarly, we found that in HaCaT cells, the levels of SPRR2A/2D transcripts were increased by RBM38, together with an increased expression of p73 (Figure 1C). Next, to determine whether SPRR2A/2D are regulated by endogenous RBM38, RBM38-KO HCT116 cells were used. As expected, RBM38 transcript and protein were undetectable along with decreased expression of p73 in RBM38-KO HCT116 cells as compared to that in isogenic control cells (Figure 1D,E), consistent with a previous report [20]. Interestingly, we found that the levels of SPRR2A/2D transcripts were also reduced in RBM38-KO HCT116 cells as compared to that in isogenic control cells (Figure 1E).

Since RBM38 regulates p73 expression, it is possible that p73 is involved in RBM38-mediated regulation of SPRR2A/2D expression. To test this, we measured SPRR2A/2D expression in inducible RBM38-expressing HaCaT cells in which p73 was transiently knocked down by siRNA. We showed that SPRR2A/2D along with p73 was induced by RBM38 in HaCaT cells transfected with control siRNA (Figure 1F). However, SPRR2A/2D expression was not induced by RBM38 in HaCaT cells in which p73 was knocked down (Figure 1F SPRR2A and SPRR2D panels), suggesting that p73 is required for RBM38-mediated SPRR2A/2D expression.

### 3.2. p73 Directly Induces SPRR2A and SPRR2D Expression via Binding to Their Promoters

**Figure 2:**
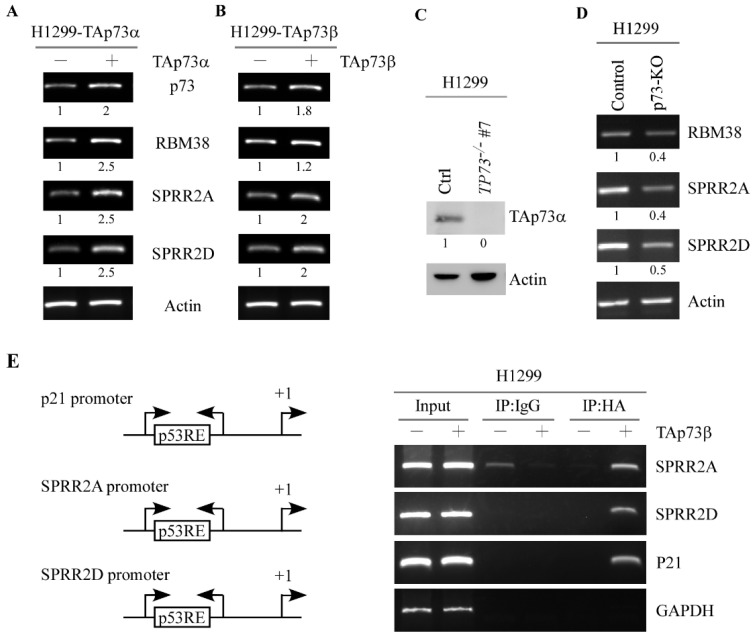
p73 directly induces SPRR2A and SPRR2D expression via binding to their promoters. (**A**,**B**) The levels of p73, RBM38, SPRR2A, SPRR2D, and actin transcripts was examined in H1299 cells that were uninduced or induced to expression TAp73α (**A**) or TAp73β (**B**) for 24 hours. (**C**) The levels of TAp73α and actin proteins were examined in isogenic control and *TP73*-KO H1299 cells by Western blot analysis. **(D)** The levels of RBM38, SPRR2A, SPRR2D, and actin transcripts were examined in in isogenic control and *TP73*-KO H1299 cells by RT-PCR analysis. (**E**) Left panel: Schematic presentation of the p21, SPRR2A, and SPRR2D promoters with the locations of potential p53-REs and PCR primers for ChIP assays. Right panel: ChIP assays were performed as described in Materials and Methods. Anti-HA antibody was used to immunoprecipitate HA-p73–DNA complexes.

To determine whether p73 directly regulates SPRR2A/2D expression, we measured the levels of SPRR2A/2D in H1299 cells that can inducibly express TAp73α or TAp73β. As shown in Figure 2A,B, the level of RBM38 transcript was increased by TAp73α or TAp73β, consistent with the previous report that RBM38 is a target of p73 [17]. We found that the levels of SPRR2A/2D transcripts were increased upon induction of TAp73α or TAp73β Figure 2A,B, SPRR2A and SPRR2D panels). To verify this, we generated H1299 cell lines in that *TP73* gene is knocked out by using CRISPR-Cas9. As expected, TAp73α protein was not detectable in *TP73^−/−^* H1299 cells as compared to that in isogenic control cells (Figure 2C, TAp73α panel). Additionally, the levels of RBM38 and SPRR2A/2D transcripts were decreased by knockout of p73 in H1299 cells (Figure 2D, RBM38, SPRR2A, and SPRR2D panels). Next, a ChIP assay was performed to determine whether p73 directly binds to the SPRR2A/2D promoters. We showed that TAp73β directly bound to the SPRR2A/2D promoters (Figure 2G, SPRR2A and SPRR2D panels). As a positive control, TAp73β was found to bind to the p21 promoter (Figure 2E, p21 panel).

### 3.3. SPRR2A and SPRR2D Are Induced by p73 and RBM38 for Keratinocyte Differentiation

**Figure 3:**
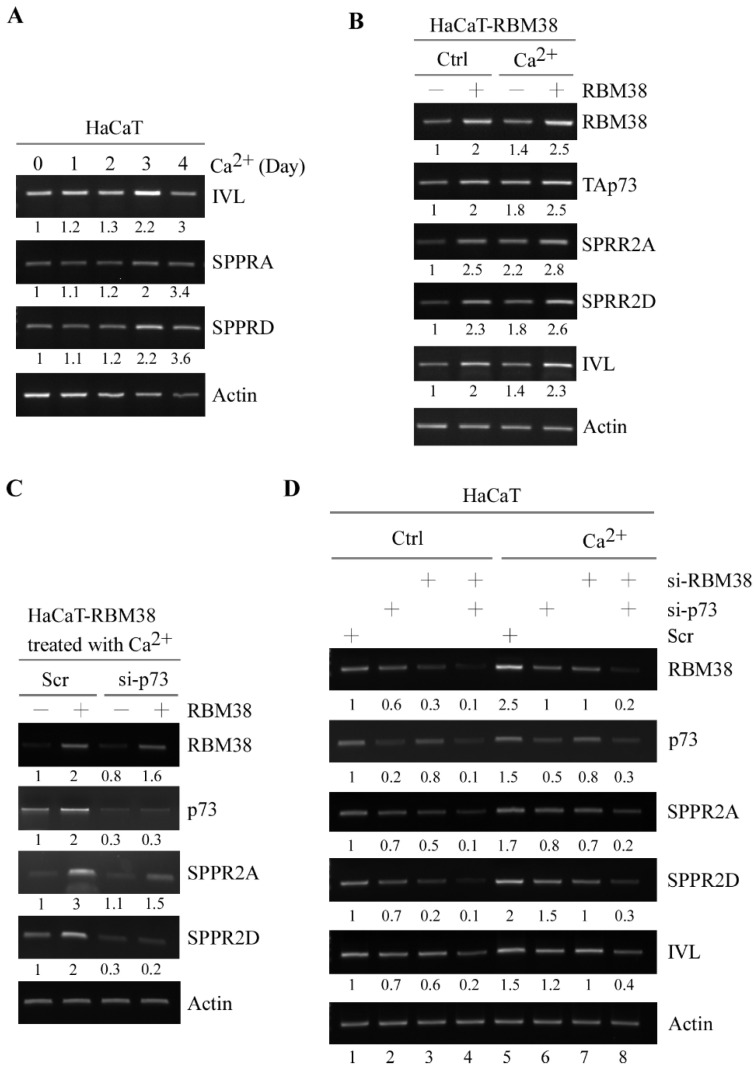
SPRR2A and SPRR2D are induced by p73 and RBM38 to promote keratinocyte differentiation. (**A**) HaCaT cells were treated with Ca^2+^ from 0–4 days and the levels of IVL, SPRR2A, SPRR2D and actin transcripts were examined by RT-PCR analysis. (**B**) HaCaT cells was uninduced or induced to express RBM38 for 12 hours, followed by treatment with or without Ca^2+^ for 3 days. The levels of RBM38, p73, IVL, SPRR2A, SPRR2D and actin transcripts were examined by RT-PCR analysis. (**C**) HaCaT cells uninduced or induced to express RBM38 were transiently transfected with a control or p73 siRNA for 24 h, followed by Ca^2+^ for 3 days. The levels of RBM38, p73, SPRR2A, SPRR2D and actin transcripts were examined by RT-PCR analysis. (**D**) HaCaT cells were transiently transfected with a control siRNA or siRNA against either p73 or RBM38 or both for 24 hours, followed with or without Ca^2+^ for 3 days. The levels of RBM38, p73, SPRR2A, SPRR2D, IVL and actin transcripts were examined by RT-PCR analysis.

As structure proteins of cornified cell envelopes, SPRR2A/2D are essential for keratinocyte differentiation [31,32]. Previously, we showed that RBM38 plays a role in keratinocyte differentiation [19]. Thus, we sought to determine whether SPRR2A/2D are involved in RBM38/p73-regulated keratinocyte differentiation. To test this, we examined whether SPRR2A/2D expression was altered during keratinocyte differentiation in HaCaT cells. We would like to mention that upon treatment with Ca^2+^, HaCaT cells undergo keratinocyte differentiation and form cornified envelopes [33,34]. As expected, the levels of SPRR2A/2D transcripts along with involucrin (IVL), a differentiation marker [35], were increased in response to Ca^2+^ treatment in HaCaT cells (Figure 3A), consistent with a previous report that SPRR2A/2D are involved in the cornified envelop formation [31]. Next, we examined whether RBM38 can enhance SPRR2/2D expression during keratinocyte differentiation. We found that the levels of TAp73, SPRR2A, SPRR2D and IVL transcripts were increased by RBM38 (Figure 3B). Interestingly, RBM38 cooperated with Ca^2+^ treatment to further increase TAp73, SPRR2A, SPRR2D and IVL (Figure 3B).

To examine whether RBM38-mediated SPRR2A/2D expression is p73-dependent, we measure the ability of RBM38 to induce SPRR2A/2D expression in p73-knockdown HaCaT cells following treatment with Ca^2+^. We showed that the coordinated induction of SPRR2A/2D by RBM38 and Ca^2+^ treatment was abrogated by p73 knockdown in HaCaT cells (Figure 3C). To further test this, we determined whether endogenous RBM38 and p73 are necessary for SPRR2A/2D and IVL expression during keratinocyte differentiation. As expected, the basal levels of SPRR2A/2D and IVL transcript were decreased by knockdown of RBM38 and/or p73 in HaCaT cells without treatment of Ca^2+^ (Figure 3D, compare lane 1 with 2–4, respectively). Additionally, the levels of SPRR2A/2D and IVL transcripts along with RBM38 and p73 were induced by Ca^2+^ treatment (Figure 3D, compare lane 1 with 5). Interestingly, the increased expression of SPRR2A/2D and IVL by treatment with Ca^2+^ was attenuated by knockdown of RBM38 or p73 alone (Figure 3D, compare lane 5 with 6–7, respectively). Moreover, combined knockdown of RBM38 and p73 abrogated the ability of Ca^2+^ treatment to induce SPRR2A/2D and IVL expression and in fact further suppressed SPRR2A/2D and IVL expression in the presence of Ca^2+^ treatment (Figure 3D, compare lane 5 with 8). Together, these data suggest that RBM38 and/or p73 are necessary for the induction of SPRR2A/2D during keratinocyte differentiation.

### 3.4. p73 and SPRR2A/2D Are Coordinately Expressed in Normal and Neoplastic Tissues

**Figure 4:**
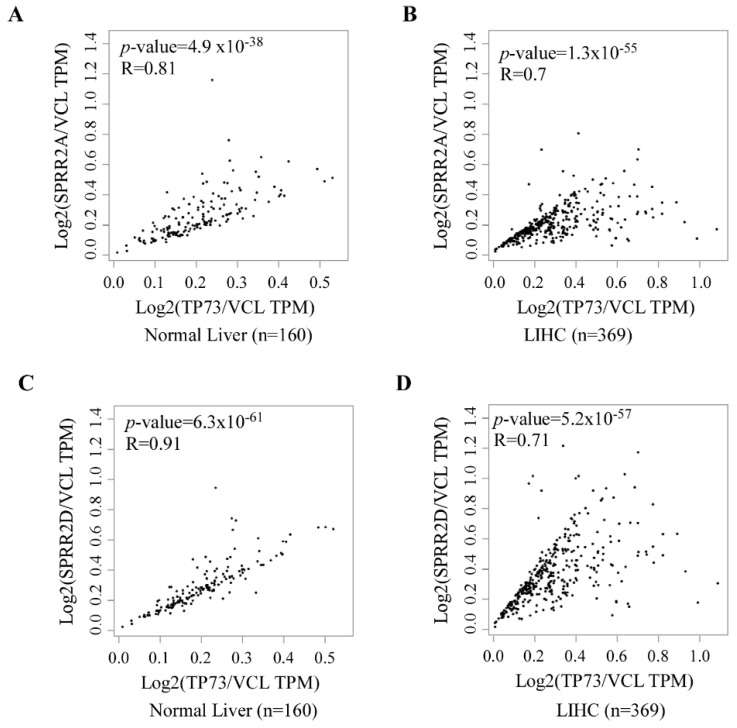
Correlation of p73 and SPRR2A/2D expression in normal and neoplastic tissues. (**A**,**B**) p73 expression is associated with SPRR2A expression in normal liver tissues (**A**) and liver hepatocellular carcinomas (**B**). The analysis was performed using the GEPIA2 database (http://gepia2.cancer-pku.cn/#correlation, accessed on 16 November 2020). Statistical analysis suggests a strong correlation between p73 and SPRR2A expression in normal liver tissues (Pearson’s r = 0.81) and cancerous liver tissues (Pearson’s r = 0.7). (**C**,**D**) p73 expression is associated with SPRR2D expression in normal liver tissues (**C**) and liver hepatocellular carcinomas (**D**). Statistical analysis suggests a strong correlation between p73 and SPRR2D expression in normal liver tissues (Pearson’s r = 0.91) and cancerous liver tissues (Pearson’s r = 0.71).

As shown above, our data suggest that SPRR2A/2D are regulated by the RBM38-p73 axis (Figure 1, Figure 2 and Figure 3). Thus, we examined whether SPRR2A/2D expression is correlated with p73 expression in normal and cancer tissues. To this end, we searched the GEPIA database (www.gepia.cancer-pku-cn, accessed on 16 November 2020). We found that the level of SPRR2A transcript was well correlated with that of p73 transcript in both normal liver (Spearman’s r = 0.81) and hepatocellular carcinomas (Pearson’s r = 0.7) (Figure 4A,B). Similarly, we found that the level of SPRR2D transcript was correlated well with that of p73 transcript in both normal liver (Spearman’s r = 0.91) and hepatocellular carcinomas (Pearson’s r = 0.71) (Figure 4C,D). Additionally, we found that the levels of SPRR2A/2D transcripts were correlated with that of p73 in both normal prostate and prostate carcinomas (Supplemental Figure S1). Together, these data suggest that SPRR2A/2D expression are controlled by p73 in both normal and neoplastic cells.

#### Loss of Rbm38 Cooperates with Trp73 Deficiency to Modulate Chronic Inflammation Potentially Via SPRR2A/2D

**Figure 5:**
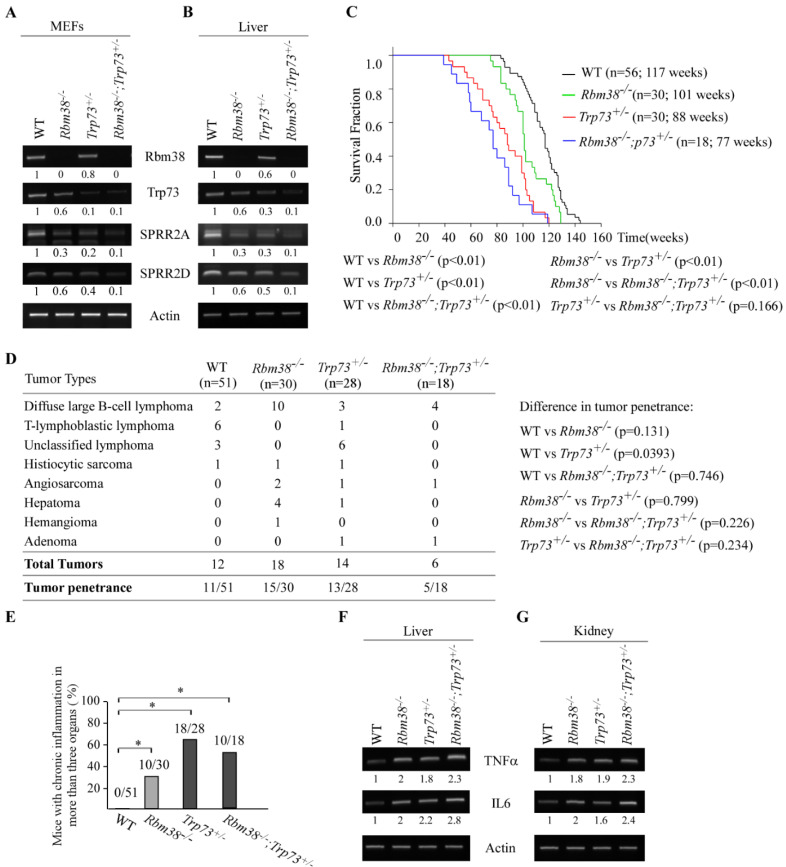
Loss of *Rbm38* cooperates with *Trp73* deficiency to modulate chronic inflammation, potentially via SPRR2A/2D. (**A**,**B**) The levels of Rbm38, Trp73, SPRR2A, SPRR2D, and actin was examined by RT-PCR analysis using WT, *Rbm38^−/−^*, *p73^+/−^*, and *Rbm38^−/−^*; *Trp73^+/−^* MEFs (**A**) and livers (**B**). (**C**) Kaplan–Meyer survival curves of WT (*n* = 56), *Rbm38^−/−^* (*n* = 30), *Trp73^+/−^* (*n* = 30), and *Rbm38^−/−^*;*Trp73^+/−^* (*n* = 18) mice. (**D**) Tumor spectrum and burden in WT (*n* = 56), *Rbm38^−/−^* (*n* = 30), *Trp73^+/−^* (*n* = 28), and *Rbm38^−/−^*;*Trp73^+/−^* (*n* = 18) mice. (**E**) The numbers and percentages of WT, *Rbm38^−/−^*, *Trp73^+/−^*, and *Rbm38^−/−^*;*Trp73^+/−^* mice with chronic inflammation in three or more organs. * indicates *p* < 0.05 (by student *t*-test). (**F**,**G**) The levels of TNFα and IL6 transcripts were examined in the livers (**F**) or kidneys (**G**) from WT, *Rbm38^−/−^*, *Trp73^+/−^*, and *Rbm38^−/−^*;*Trp73^+/−^* mice.

To understand the biological significance of the RBM38-p73 feedforward loop potentially via SPRR2A/2D in vivo, we generated a cohort of WT, *Rbm38^−/−^*, *Trp73^+/−^*, and *Rbm38^−/−^;Trp73^+/−^* MEFs. We showed that the *Rbm38* transcript was undetectable in *Rbm38^−/−^* and *Rbm38^−/−^;Trp73^+/−^* livers (Figure 5A, Rbm38 panel). Additionally, the level of *Trp73* transcript was decreased in *Rbm38^−/−^* or *Trp73^+/−^* MEFs, which was then further decreased in *Rbm38^−/−^;Trp73^+/−^* MEFs (Figure 5A, Trp73 panel). These data were consistent with our previous report that Rbm38 is required for p73 expression [20]. Importantly, we found that the levels of SPRR2A/2D transcripts were decreased in *Rbm38^−/−^* or *Trp73^+/−^* MEFs, which were further decreased in *Rbm38^−/−^;Trp73^+/−^* MEFs (Figure 5A). To validate this, compound *Rbm38^−/−^;Trp73^+/−^* mice together with WT, *Rbm38^−/−^*, and *Trp73^+/−^*mice were generated and used to examine the levels of SPRR2A/2D transcripts. We showed that the levels of SPRR2A/2D transcripts were decreased in *Rbm38^−/−^* or *Trp73^+/−^* mouse livers, which were further decreased in *Rbm38^−/−^;Trp73^+/−^* mouse liver (Figure 5B). Next, to understand the biological significance of the RBM38-p73 loop in vivo, a cohort of WT, *Rbm38^−/−^*, *Trp73^+/−^* and *Rbm38^−/−^;Trp73^+/−^* mice were generated and monitored for their lifespan, susceptibility to spontaneous tumors, chronic inflammation, and other pathological abnormalities. We would like to mention that in order to minimize the number of animals used, the survival and tumor data for WT mice had been previously reported [36,37]. For *Rbm38^−/−^* mice, 7 of them were generated for this study and 23 of them were generated previously [22]. For *Trp73^+/−^* mice, 4 of them were generated for this study and 28 were generated previously [21]. All the mice were derived from the same C57BL/6 background and maintained in the same animal facility. The median lifespan was 117 weeks for WT mice, 101 weeks for *Rbm38^−/−^* mice, 88 weeks for *Trp73^+/−^* mice, and 77 weeks for *Rbm38^−/−^;Trp73^+/−^* mice (Figure 5C and Supplementary Tables S1–S4). The lifespan was significantly shorter for *Rbm38^−/−^*, *Trp73^+/−^* and *Rbm38^−/−^;Trp73^+/−^* mice than that for WT mice (Figure 5C). Additionally, the lifespan for *Trp73^+/−^* or *Rbm38^−/−^;Trp73^+/−^* mice was shorter than that for *Rbm38^−/−^* mice. However, although the median lifespan for *Rbm38^−/−^;Trp73^+/−^* was 11-week shorter than that for *Trp73^+/−^* mice, the difference was not statistically significant, probably due to a few mice with relatively long lifespans (Figure 5C). Next, histological analysis was performed and showed that spontaneous tumors occurred in 11 out of 51 wild-type mice, 15 out of 30 *Rbm38^−/−^* mice, 13 out of 28 *Trp73^+/−^* mice, and 5 out of 18 *Rbm38^−/−^;Trp73^+/−^* mice (Figure 5D). Fisher’s exact test showed that the tumor incidence was significantly higher in *Trp73^+/−^* or *Rbm38^−/−^* mice than that in WT mice (WT vs. *Trp73^+/−^*, *p* = 0.0393; WT vs. *Rbm38^−/−^*, *p* = 0.0131), consistent with previous reports [21,22]. While the percentage of *Rbm38^−/−^;Trp73^+/−^* mice (27.8%) with spontaneous tumors were higher than that of WT mice (21.5%), the difference was not statistically significant (Figure 5D). Similarly, while *Trp73^+/−^* or *Rbm38^−/−^* mice developed more numerous spontaneous tumors than *Rbm38^−/−^;Trp73^+/−^* mice, the rate of tumor incidence between *Rbm38^−/−^;Trp73^+/−^* and *Trp73^+/−^* or *Rbm38^−/−^* mice was not statistically significant (Figure 5D).

Since *Trp73^+/−^* and *Rbm38^−/−^* mice were prone to chronic systemic inflammation, that is, chronic inflammation in three or more organs [21,22], we sought to determine whether compound *Rbm38^−/−^;Trp73^+/−^* mice are also susceptible to chronic systemic inflammation. We found that systemic inflammation was detected in 0 out of 51 WT mice, 10 out of 30 *Rbm38^−/−^* mice, 18 out of 28 *Trp73^+/−^* mice, and 10 out of 18 *Rbm38^−/−^;Trp73^+/−^* mice. Statistical analysis showed that similar to that in *Trp73^+/−^* or *Rbm38^−/−^* mice, the percentage of mice with systemic inflammation was much higher in *Rbm38^−/−^;Trp73^+/−^* mice than that in WT mice (Figure 5E). To further test this, the level of two pro-inflammatory cytokines, such as TNFα and IL-6, were examined in liver and kidney tissues from age- and gender-matched WT, *Rbm38^−/−^*, *Trp73^+/−^* and *Rbm38^−/−^;Trp73^+/−^* mice. We found that the levels of TNFα and IL-6 transcripts were much higher in the livers or kidneys from *Rbm38^−/−^*, *Trp73^+/−^* and *Rbm38^−/−^;Trp73^+/−^* mice than that in the WT mice (Figure 5F,G), consistent with the extent of systemic inflammation observed in these mice (Figure 5E). Together, these data suggest that deficiencies in Rbm38 and p73 result in weak induction of barrier-protecting genes, such as SPRR2A/2D, leading to early death (shortened lifespan) in *Rbm38^−/−^;Trp73^+/−^* mice.

## 4. Discussion

RBM38 and p73 forms a feedforward regulatory loop [17,20]. However, the biological significance of the RBM38-p73 loop remains to be elucidated. Here, we found that SPRR2A/2D are bona fide targets of p73 and that RBM38-mediated expression of SPRR2A/2D is p73-dependent (Figure 1 and Figure 2). We also found that induction of SPRR2A/2D during keratinocyte differentiation is dependent on both p73 and Rbm38 (Figure 3). Similarly, SPRR2A/2D expression is found to correlate well with p73 expression in normal and cancerous tissues (Figure 4). Conversely, SPRR2A/2D expression is decreased by lack of *Rbm38* or *Trp73*, which is further decreased by deficiencies in both *Rbm38* and *Trp73* (Figure 5). Interestingly, *Rbm38^−/−^;Trp73^+/−^* mice had a shortened lifespan and were highly susceptible to systemic chronic inflammation (Figure 5). Together, our data suggest that SPRR2A/2D are regulated by the RBM38-p73 axis, which contributes to p73-mediated suppression of inflammatory response.

SPRR proteins belong to one of the subfamilies of the epidermal differentiation complex (EDC), including late cornified envelope (LCE), filaggrin (FLG) and FLG-like (FLG-like), and S100 proteins [38,39]. These EDC proteins are coordinately expressed during epidermal differentiation and critical for the formation and maintenance of the epidermal cornified cell envelope, an effective barrier against both internal and external insults [29,39]. Indeed, studies showed that disruption of the epithelial barrier leads to increased permeability of the epithelial barrier and subsequently inflammatory diseases [40,41]. Moreover, alteration of SPRRs occurs in many inflammatory disorders [42], such as psoriatic epidermis and epidermolytic hyperkeratosis, as well as in chronic inflammation [30], such as eosinophilic esophagitis. Here, we found that SPRR2A/2D expression was markedly suppressed along with heightened inflammatory response in *Rbm38^−/−^;Trp73^+/−^* mice (Figure 5). Considering that *Rbm38^−/−^;Trp73^+/−^* mice had a shortened lifespan and were prone to chronic inflammation (Figure 5), we postulated that decreased expression of SPRR2A/2D in *Rbm38^−/−^;Trp73^+/−^* mice resulted in loss of the epithelial barrier, leading to chronic systemic inflammation and ultimately early death (shortened lifespan).

Despite the fact that chronic inflammation is closely related to cancer development, we did not observe enhanced tumor penetrance in *Rbm38^−/−^;Trp73^+/−^* mice (Figure 5D). In fact, although *Rbm38^−/−^;Trp73^+/−^* mice developed more numerous spontaneous tumors than WT mice, the difference was not statistically significant (Figure 5D). Similarly, while *Trp73^+/−^* or *Rbm38^−/−^* mice developed more numerous spontaneous tumors than *Rbm38^−/−^;Trp73^+/−^* mice, the rate of tumor incidence between *Rbm38^−/−^;Trp73^+/−^* and *Trp73^+/−^* or *Rbm38^−/−^* mice was not statistically significant (Figure 5D). The phenotype is surprising but not totally unexpected for the following reasons. First, it is possible that RBM38 and p73 regulate two divergent pathways involved in chronic inflammation vs. tumor suppression. Indeed, *Rbm38^−/−^* mice had a longer lifespan and were less prone to chronic inflammation than *Trp73^+/−^* mice (Figure 5). Second, it is possible that reduced p73 expression in *Rbm38^−/−^;Trp73^+/−^* mice suppresses p73-dependent transcriptional program necessary for suppression of chronic inflammation, such as SPRR2A/2D. Thus, chronic systemic inflammation observed in *Rbm38^−/−^;Trp73^+/−^* mice may be responsible for early death and shortened lifespan, which precedes tumor development. Thus, further studies are warranted to determine whether SPRR2A/2D expression mediated by the RBM38-p73 axis links chronic inflammation and tumor suppression.

We found that SPRR2A/2D are induced by RBM38 and p73 during keratinocyte differentiation (Figure 3), suggesting that RBM38 and p73 play a role in cornification. Recently, it was found that SPRR1B, another member of small proline-rich family proteins, is regulated by p73 during skin development and wound healing [43]. SPRR1B is closely related to SPRR2A/2D. Thus, it would be interesting to determine whether the RBM38-p73 axis plays a role in skin development, cornification and wound healing via SPRR2A/2D.

## 5. Conclusion

In this work, we showed that SPRR2A and 2D are bona fide targets of p73 and that RBM38-mediated expression of SPRR2A/2D is p73-dependent. We also found that SPRR2A and SPRR2D are involved in p73-mediated keratinocyte differentiation and possibly, chronic inflammation. Further, SPRR2A/2D expression is found to correlate well with p73 expression in normal and cancerous tissues. Together, these data suggest that SPRR2A/2D are regulated by the RBM38-p73 axis and contributes to p73-mediate tumor suppression and inflammatory response.

## Data Availability

The data presented in this study are available on request from the corresponding authors.

## Supplementary Materials

The following are available online at https://www.mdpi.com/article/10.3390/cancers13112829/s1, Figure S1: Correlation of p73 and SPRR2A/2D expression in normal prostateand prostate carcinomas, Table S1: Wild type (WT) mice (*n* = 56)—survival time, tumor spectrum, steatosis, inflammation, and other abnormalities, Table S2: *Rbm38^−/−^*mice (*n* = 30)—survival time, tumor spectrum, inflammation, and other abnormalities, Table S3: *Trp73^+/−^*mice (*n* = 32)—survival time, tumor spectrum, steatosis, inflammation, and other abnormalities, Table S4: *Rbm38^−/−^*; *Trp73^+/−^*mice (*n* = 18)—survival time, tumor spectrum, steatosis, inflammation, and other abnormalities.

## Abbreviations

SPRR2A: small proline-rich protein 2A; SPRR2D, small proline-rich protein 2D; KO, knockout; 3′UTR, 3′ untranslated region; CRISPR, clustered regularly interspaced short palindromic repeats; EDC, epidermal differentiation complex; LCE, late cornified envelope; FLG, filaggrin; IVL, involucrin; MEF, mouse embryonic fibroblast; WT, wild type.
