# Supplementary material for: Small Proline-Rich Protein 2A and 2D Are Regulated by the RBM38-p73 Axis and Associated with p73-Dependent Suppression of Chronic Inflammation
Source: Cancers (Basel). 2021 Jun 6;13(11):2829. doi: 10.3390/cancers13112829 (PMC8201237; doi:10.3390/cancers13112829)
Supplement: Supplementary file 1 [file cancers-13-02829-s001.zip › cancers-1195332-supplementary.pdf]

Supplemental Table S1: Wild type (WT) mice (n=56) - survival time, tumor spectrum, steatosis, inflammation, and other abnormalities

ID	Gender	Survival (Wks)	Tumor	Inflammation	Other abnormalities
5	F	134	No	No	No
7	F	117	No	No	No
16	F	100	No	No	No
22	F	109	No	No	No
25	F	109	No	No	No
44	F	90	No	No	No
55	F	104	T-LBL	No	No
64	F	120	No	No	No
2	M	127	No	No	No
3	M	117	No	Liver	No
12	M	127	No	No	No
13	M	127	No	No	No
20	M	122	No	No	No
23	M	122	No	No	No
26	M	127	No	Liver/Salivary gland	No
34	M	124	DLBCL	No	No
37	M	134	No	No	No
62	M	128	No	No	No
45	M	133	No	No	No
49	M	117	No	No	No
50	M	113	T-LBL/ DLBCL	No	No
56	M	117	DLBCL	No	No
59	M	119	DLBCL	No	No
65	M	106	No	No	No
69	M	102	DLBCL	No	Spleen hyperplasia
70	M	103	No	No	Thymus hyperplasia
71	M	90	No	No	No
1-24-2	M	83	No	No	No
2-15-2	F	140	Lymphoma	No	EMH in liver
2-19-6	M	132	No	Pancreas	EMH in Spleen
2-19-2	F	143	No	No	EMH in Spleen
3-11-3	M	129	No	No	No
3-28-5	F	85	No	No	EMH in spleen
3-9-7	M	129	No	No	No
5-12-3	F	99	Lymphoma	No	EMH in Spleen
7-9-9	F	116	No	No	No
8-2-6	M	120	No	No	No
10-24-7	F	130	No	Pancreas	EMH in spleen
10-26-6	F	129	Histiocytic sarcoma	No	EMH in spleen/liver
11-10-7	M	121	No	No	No
11-7-3	F	121	No	No	No
11-29-2	F	144	No	No	EMH in spleen
12-2-4	F	113	No	No	No
12-20-7	F	115	No	No	EMH in Spleen
11-9-6	F	96	DLBCL	Skin	No
1-19-1	F	86	No	Skin	No
11-10-15	F	105	No	Skin/Pancreas	No
12-25-6	M	111	No	No	Hepatocirrhosis
1-19-5	F	109	Lymphoma	No	No
7-22-4	M	86	No	No	No
7-22-7	M	126	No	Kidney	No
11	M	111	N/A		Found dead
42	M	111	N/A		Found dead
43	M	107	N/A		Found dead
46	M	117	N/A		Found dead
52	M	101	N/A		Found dead

These mice were from published studies (Yang et al, 2017, PNAS, 114 (43) 11500-11505; Zhang et al, 2017, Genes & Dev, 31:1243-56)
T-LBL: Thymic lymphoblastic lymphoma; DLBCL: Diffuse large B-cell lymphoma; N/A: not applicable; EMH: extramedullary hematopoiesis

Supplemental Table 2 : Rbm38$^{-/-}$ mice (n=30) - survival time, tumor spectrum, inflammation, and other abnormalities

ID	Gender	Survival (Wks)	Tumor	Inflammation	Other abnormalities
1-12-2	M	100	Lymphoma	Pancreas/Lung/Liver/Salivary gland	EMH in spleen
1-24-5	M	83	-	Lung/Liver	-
1-11-3	M	100	-	Lung	-
1-11-14	F	110	Histiocytic sarcoma	Pancreas/Lung/Liver/Salivary gland/Kidney	EMH in spleen
2-14-2	M	109	-	Lung	EMH in spleen
2-14-3	M	101	-	-	EMH in spleen
2-25-1	F	107	Lymphoma	-	EMH in spleen
3-7-2	F	101	Lymphoma	Lung/Liver	EMH in spleen
3-7-5	M	129	Lymphoma	Kidney/Liver	EMH in liver
3-7-4	M	129	Hepatoma	Pancreas/Kidney/Liver	EMH in spleen
6-15-3	F	90	-	Liver/Lung	EMH in spleen and liver;Liver steatosis
8-2-3	F	94	-	Kidney	EMH in spleen
9-30-4	M	102	Hemangiosarcoma	Pancreas/Lung/Liver	EMH in spleen and liver
10-26-3	F	75	-	Pancreas/Lung	EMH in spleen
11-3-14	M	77	-	-	EMH in spleen
11-10-10	M	124	Lymphoma and hemangiosarcoma	Pancreas/Lung/Kidney/Salivary gland	EMH in spleen and liver
11-16-14	M	128	-	Lung	EMH in spleen and liver
11-16-7	F	125	Hepatoma and lymphoma	Pancreas/Lung/Liver/Salivary gland	EMH in spleen
12-19-3	M	123	Lymphoma	Salivary gland	EMH in spleen
12-19-8	M	118	Hepatoma	Pancreas	EMH in spleen and liver
12-19-6	F	87	-	-	-
12-24-2	M	122	-	Liver	Liver steatosis
12-28-5	F	102	Hepatoma and lymphoma	Pancreas/Lung/Liver/Salivary gland	EMH in spleen
5-13-1+2	M	83	-	Lung/Salivary gland	-
11-26-1	F	100	-	Kidney/Lung/Salivary gland	EMH in spleen, Lymphoid hyperplasia
8-31-6	M	100	-	Kidney;Lung	EMH in spleen
7-16-1	M	83	-	Kidney/Lung/Liver/Salivary gland	EMH in spleen, Liver steatosis
6-17-2	M	95	Lymphoma	Kidney	EMH in spleen
6-9-3	F	96	Lymphoma	Salivary gland	EMH in spleen, Lymphoid hyperplasia
5-13-6	M	101	Hemangioma	Kidney/Lung/liver/Salivary gland	EMH in spleen, Steatosis

The data of the first 23 mice were from published studies (Zhang et al, 2014, PNAS, 111 (52) 18637-18642)
EMH: extramedullary hematopoiesis

Supplemental Table S3 : Trp73$^{+/-}$ mice (n=32)- survival time, tumor spectrum, steatosis, inflammation, and other abnormalities

ID#	Gender	Survival (Wks)	Tumor	Inflammation	Other abnormalities
1-30-11	F	82	-	Liver/Salivary gland/ kidney/ pancreas	SWPH/ EMH
6-7-14	M	43	-	Liver/lung/Kidney/ Salivary gland/skin	SWPH / EMH / TH
9-23-1	F	77	Hepatoma	Liver/pancreas/kidney	SWPH
12-14-3	F	46	-	Skin/lung/kidny/liver/salivary gland	SWPH / EMH
5-27-14	M	65	-	Liver/salivary gland	SWPH / EMH
9-13-11	F	69	-	Liver/Salivary gland/lung/kidney	SWPH / EMH
11-6-1	F	55	T-LBL	Liver/Kidney/Salivary gland/pancreas/lung	SWPH / EMH
5-3-5	F	105	-	kidney/liver/pancreas/salivary gland	SWPH / EMH / TH/ Steatosis
11-6-5	F	88	DLBCL/Hemangioma	Liver/Salivary gland	SWPH / EMH / TH/ Steatosis
5-13-2	M	117	-	Liver/Salivary gland/Kidney	SWPH / EMH / TH/ Steatosis
11-13-1/5	M	99	-	Liver, Salivary gland	-
10-13-12	F	89	Lymphoma	-	-
11-5-7	F	102	DLBCL/Lymphoma	Kidney/Liver/Cecum	SWPH
6-11-6	M	108	Lymphoma	Liver	SWPH
10-3-11	F	99	-	Liver/salivary gland/lung	SWPH / TH
10-22-4	F	86	Histiocytic sarcoma	Kidney/Salivary gland	-
5-28-16	F	120	Lymphoma	-	-
11-13-4	F	74	Lymphoma	-	-
12-14-1	F	103	-	Liver/pancreas/Salivary gland	SWPH
1-25-4	F	94	DLBCL	-	-
1-15-8	F	69	-	Liver/Kidney/salivary gland	SWPH
10-31-4	F	88	Lymphoma	Liver/Kidney/Lung/Slivary gland	SRPH / EMH
10-14-3/4	F	26	-	Liver/Kidney/lung/Salivary gland	SRPH / EMH/ Steatosis
11-6-4	F	62	Gastric Adenoma	Lung/Salivary gland/Liver/Kidney	SRPH/ EMH
9-16-5	F	76	-	Kidney/Liver/Salivary gland	SRPH/EMH<br>Liver multifocal necrosis
6-22-4	F	80	-	Kidney/Lung/liver	SRPH/EMH<br>Liver multifocal necrosis
1-2-5	M	7			Hydrocephalus
11-13-2	M	11	-		Hydrocephalus
4-19-1	F	102	-	Liver/Heart	SWPH/Follicular hyperplasia
10-31-5	F	99	DLBCL	Kidney/Lung/Salivary gland	Steatosis
10-9-2	M	101	N/A		Found dead
3-10-3	M	108	N/A		Found dead

The data of the first 28 mice were from published studies (PNAS, 2019;116(48):24259-24267; J Pathol 2020; 251:284–296)
DLBCL:Diffuse large B-cell lymphoma; EMH: Extramedullary hematopoiesis;
SWPH: Spleen white pulp hyperplasia; SRPH: Spleen red pulp hyperplasia; TH: Thymic hyperplasia
DLBCL: Diffused Large B-cell lymphoma

Supplemental Table S4: Rbm38$^{-/-}$;Trp73$^{+/-}$ mice (n=18)- survival time, tumor spectrum, steatosis, inflammation, and other abnormalities

ID	Gender	Survival (Wks)	Tumor	Inflammation	Other abnormalities
11-6-3	F	119	No	Kidney	EMH/TH
8-10-4	F	68	No	salivary gland	EMH/TH
7-27-6	F	58	Lymphoma	Kidney/Lung	EMH
9-7-3	F	89	Lymphoma	Liver/Kidney/Lung/salivary gland	EMH
6-10-3	F	59	Lymphoma	Kidney/Liver/Salivary gland	EMH/TH
9-7-2	F	89	No	Kidney/pancreas/salivary gland	EMH/TH
4-26-9	F	49	No	Kidney/salivary gland/liver/kidney	EMH/TH
3-18-5	F	80	No	Salivary gland/GI tract	EMH/TH/white pulp atrophy
5-13-11	F	74	No	Kidney/Lung/salivary gland	EMH
10-9-1	F	107	Adenocarcinoma/ Hemangioma	Liver/Kidney/lung	EMH/SWHP
9-7-1	M	39	No	Salivary gland	EMH/TH
12-8-4	F	77	Lymphoma	Kidney/salivary gland	EMH
11-26-4	F	60	No	Liver/Kidney/Lung/salivary gland	EMH/SWHP/TH
6-17-3	M	45	No	Kidney/salivary gland	EMH/SWHP/TH
3-17-1	M	92	No	Kidney/Lung/liver/salivary gland	EMH
8-31-2	M	86	No	Kidney/Lung/Salivary gland	EMH/SWHP/Steatosis
6-9-5	F	97	No	Liver/Kidney/Lung/salivary gland	EMH/SWHP
10-16-7	F	77	No	Liver/Lung/Salivary gland	EMH/SWHP

EMH: extramedullary hematopoiesis; SWHP: Spleen White Pulp Hyperplasia; TH: Thymic Hyperplasia

Supplemental Fig. 1

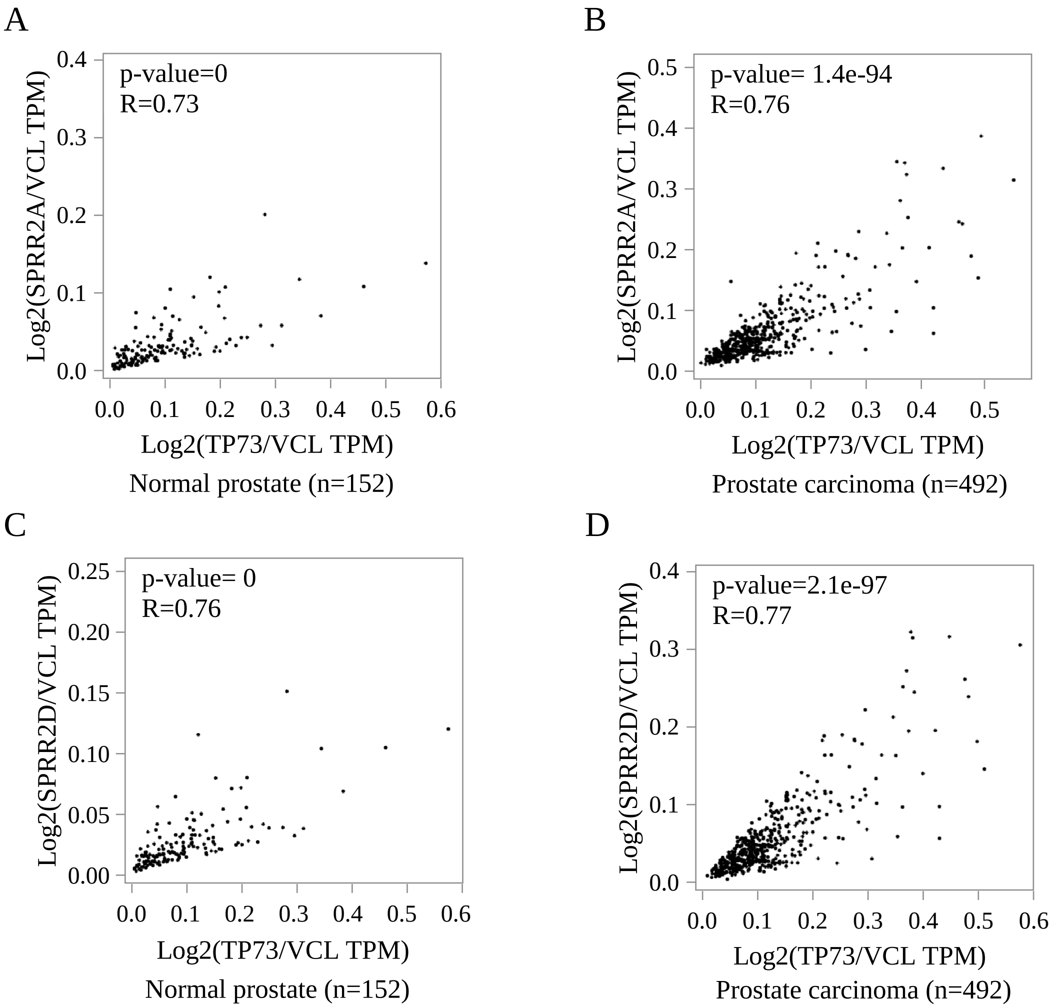

Supplemental Figure 1 Correlation of p73 and SPRR2A/2D expression in normal prostate and prostate carcinomas

(A-B) p73 expression is associated with SPRR2A expression in normal prostate tissues (A) and prostate carcinomas (B). Statistical analysis suggests a strong correlation between p73 and SPRR2A in normal liver tissues (Pearson‘s r = 0.73) and prostate carcinomas (Pearson’s r = 0.76).

(C-D) p73 expression is associated with SPRR2D expression in normal prostate tissues (C) and prostate carcinomas (D). Statistical analysis suggests a strong correlation between p73 and SPRR2D in normal prostate tissues (Pearson‘s r = 0.76) and prostate carcinomas (Pearson’s r = 0.77).
